# Synthesis and Characterization of Polyacetylene with Side-chain Thiophene Functionality

**DOI:** 10.3390/ijms9030383

**Published:** 2008-03-18

**Authors:** Banu Koz, Baris Kiskan, Yusuf Yagci

**Affiliations:** Istanbul Technical University, Department of Chemistry, Maslak 34469, Istanbul, Turkey Tel. +90 212 285 6325 or 3241; Fax: +90 212 285 6169 or 6386; E-mails: banukoz@itu.edu.tr; kiskanb@itu.edu.tr; yusuf@itu.edu.tr

**Keywords:** Polyacetylene, Helical polymer, Conjugated polymer, Thiophene

## Abstract

A new polyacetylene derivative with electroactive thiophene substituent, namely poly(2-methylbut-2-enyl thiophene-3-carboxylate) was synthesized and characterized. For this purpose, novel acetylene monomer was synthesized by the reaction of 3-thiophenecarboxylic acid with propargyl bromide and polymerized with a Rh catalyst to give the corresponding polymer. The chemical structure of the polymer was characterized to comprise the conjugated backbone and electroactive thiophene side group. UV spectral changes of the polymer with temperature were also studied. The polymer exhibited better thermal stability than the unsubstituted polyacetylenes.

## Introduction

It is known that substituted acetylenes polymerize with transition metal catalysts [1–4]. Among various catalysts used, Rh based catalysts received particular interest as they efficiently polymerize mono-substituted acetylenes, especially phenylacetylene [4–12]. Rh catalysts are also capable of polymerizing monomers with polar substituents such as propiolic esters [13–18] and propargyl amide [19–22]. Moreover, polymerization is tolerant to protic solvents such as alcohols [5, 7], amines [8], and even water [10] and ionic liquids [9] and selectively give stereo-regular polymers with cistransoid isomer having helical main chain [4–6]. Providing that the helical sense of the π-conjugated polymers is controlled, the polymer backbone becomes optically active [23–26]. The backbone chirality of the π-conjugated polymers can be detected directly by measuring their CD behavior, since the main-chain itself is a chromophore. Meanwhile, substituted polyacetylenes exhibit unique properties such as semi-conductivity, nonlinear optical properties, and high gas permeability due to the conjugated main chain and rigid molecular structure [1, 27–29]. However, notoriously intractable and thermally unstable nature of polyacetylenes is deterrent for their potential use in technological applications. Attachment of aromatic pendants to the polyacetylene backbone is one way to overcome problems associated with intractability and thermal degradation [4, 30–38]. For example, poly-(1-phenyl-1-alkyne)s are soluble in common solvents and do not decompose at elevated temperatures for a prolonged period of time [35] It is expected that incorporation of various substituents to acetylenes and their subsequent polymerization may lead to the conjugated polymers with new properties. Polymers containing thiophene units have been the subject of extensive research for more than 25 years. Polythiophenes are interesting for their not only electrical properties, but also electrophysical, magnetic, liquid crystalline and optical properties [39, 40]. However, polythiophenes suffer from the poor mechanical and physical properties. These properties can be improved by incorporating thiophene moieties into other insulating polymers and subsequent polymerization through these electroactive thiophene groups [41–43]. Various controlled [44–47] and conventional [48] polymerization methods to incorporate thiophene groups into polymers have recently been reported. It seemed therefore appropriate to synthesize acetylene with electroactive thiophene group. The corresponding polymers may form helical thiophene strands as well as a helical polyacetylene main chain possessing unique electronic and photonic functions. In this study, we report synthesis of acetylene with side-chain thiophene moiety and its polymerization with Rh catalyst in conjunction with co-catalyst. Structural, thermal and electrochemical characterizations of the monomer and corresponding polymer were performed by FT-IR, ^1^H-NMR, UV, TGA and CV measurements.

## Results and Discussions

**Scheme 1.**
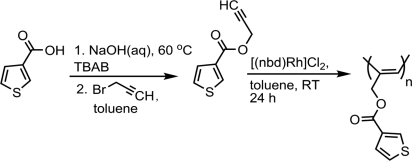
Synthesis and Polymerization of Propargyl-thiophene by Using Rh(nbd)Cl_2_].

The synthetic strategy used to prepare propargyl thiophene, as monomer, based on heterogeneous esterification reaction between 3-thiophenecarboxylic acid and propargyl bromide in basic medium (Scheme 1).

**Figure 1.**
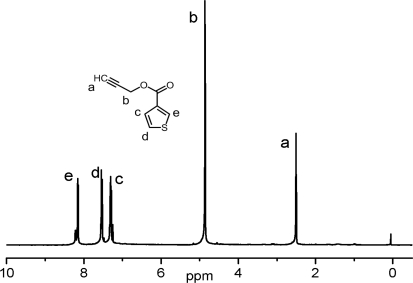
^1^H NMR spectrum of propargyl-thiophene.

The chemical structure of propargyl thiophene was confirmed by both FT-IR and ^1^H-NMR spectroscopy. As can be seen from Figure 1, ^1^H-NMR spectrum exhibits structural characteristics of both acetylene and thiophene units. The signal of terminal acetylene proton emerges as triplet at 2.50 ppm with 2.4 Hz *J*, and the two **C3** protons of the propargyl part were noted as a doublet at 4.86 ppm with 2.5 Hz *J*. Additionally, **C2, C4** and **C5** protons of thiophene heterocycle appear at 8.16 ppm as doublet of doublet (dd) with ^4^*J*_13_: 3 Hz and ^5^*J*_14_: 1.3 Hz, at 7.30 ppm as dd, ^3^J_34_: 5.9 Hz and ^4^*J*_13_: 3 Hz, at 7.53 ppm as dd, ^3^*J*_34_: 5.2 Hz and ^5^*J*_14_: 1.3 Hz, respectively.

**Figure 2.**
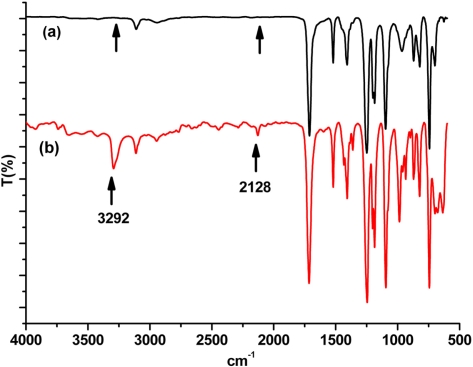
FT-IR spectra of (a) PAT-2 and (b) propargyl-thiophene.

The FT-IR spectrum shown in Figure 2 (b) also establishes the structure of the monomer. Accordingly, diagnostic stretching vibrations of ester carbonyl, aromatic C-H and terminal acetylenic C-H and C ≡ C bands appear at 1716 cm^−1^, 3112 cm^−1^, 3292 cm^−1^ and 2128 cm^−1^, respectively. Moreover, sp^2^ C-O and sp C-O stretching vibrations observed at 1246 and 1095 cm^−1^ are additional support for the ester structure.

Propargyl thiophene is expected to undergo polymerization with Rh catalyst through the acetylenic group as depicted in Scheme 1. The Rh-catalyzed polymerization reaction in toluene proceeded smoothly at ambient temperature and gave the expected polyacetylene in moderate yields after precipitation. In this polymerization, (bicyclo[2,2,1]hepta-2,5-diene)chlororhodium(I) dimer, abbreviated as [(nbd)RhCl]_2_, was selected as the catalyst due to its widespread use in related polymerizations. The results of polymerizations under different experimental conditions are given in Table 1.

As can be seen, polymerization with all co-catalysts used resulted in polymers with relatively low yields and molecular weights. Limited chain growth is probably due to the inefficient ligation of co-catalysts and monomer together to the growing species [49, 50]. The chemical structure of the polyacetylene obtained was confirmed by both FT-IR and ^1^H-NMR spectroscopy. In the FT-IR spectrum (Figure 2), the disappearance of the acetylenic C-H and C ≡ C stretching vibrations at 3292 cm^−1^ at 2128 cm^−1^, respectively, was clearly noted. Also, carbonyl C=O stretching at 1716 cm^−1^ and sp^2^ C-O and sp C-O stretching vibrations at 1246, 1095 cm^−1^ are evidencing the retention of ester group after the polymerization.

**Figure 3.**
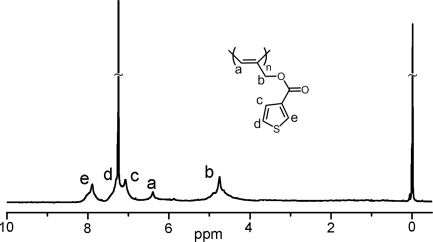
^1^H NMR spectrum of polymer PAT-2.

Further analysis of the polymer by ^1^H-NMR as presented in Figure 3 indicated the characteristic peak for cisoid =C-***H*** proton at 6.4 ppm. Additionally, the two protons, neighboring ester group and double bond emerge at 4.75 ppm with a slight shift compared to **C3** protons of the precursor propargyl unit (see Figure 1). This shift clearly suggests the transformation of triple bond to double bond. The retention of aromatic peaks was also noted.

Electrochemical property of the polymer was investigated by cyclic voltammetry (CV). Reversible redox potentials and LUMO energy values based on the value of 4.8 eV for ferrocene (F_C_) with respect to zero vacuum level [51, 52] were determined and summarized in Table 2. As can be seen poly(acetylene-thiophene) displays two cathodic peaks and two anodic peaks. The reduction potentials are 0.71 V and 1.16 V and LUMO is 4.09 eV. These results clearly indicate the electroactivity of the polymer. It is worth to mention that no detectable redox peaks were observed with the polymers possessing non-conjugated backbone i.e., methacrylate and maleimide polymers with side chain thiophene unit [42–43]. However, they become electroactive only in the presence of bare monomers such as thiophene and pyrrole. The enhanced activity in our case may be due to the conjugated backbone. In this connection, it should be pointed out that polyacetylenes with directly attached thiophene units were previously reported. However, no information on their electrochemical properties was given [53].

**Figure 4.**
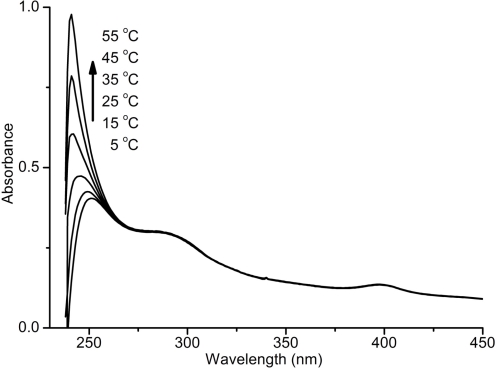
UV-vis spectral changes of PAT-2 from 5 to 55 °C measured in CHCl_3_ [PAT-2]= 1.8 × 10^−5^.

**Figure 5.**
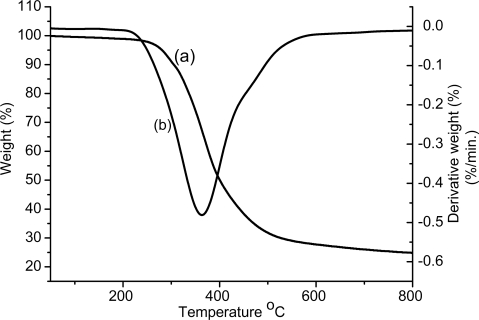
TGA thermogram of PAT-2 (a) recorded under nitrogen at a heating rate of 10 °C/min., (b) derivative of curve (a).

Figure 4 shows the UV spectral changes of the polymer solution in CHCl_3_ with temperature. As can be seen, the absorbance at lower wavelengths increases by increasing the temperature probably due to the transformation to a non-ordered structure. Thermal stability of the poly(acetylene-thiophene) (PAT-2) was investigated by thermal gravimetric analysis (TGA) under nitrogen exposure. The TGA profile of the polymer is shown in Figure 5 and the results are summarized in Table 3. It is well known that monosubstituted polyacetylenes are generally thermally unstable. Typically, poly(1-hexyne) starts to lose its weight at ∼ 150 °C. Interestingly, the temperature for 5% weight loss is 230 °C for PAT-2. In fact, this value is slightly lower than that of the another aromatic substitutued poly(phenyl acetylene) (T= ∼ 264 °C) [35–38].

In conclusion, a new conjugated polymer, polyacetylene, with electroactive active thiophene groups was synthesized by using a Rh catalyst and characterized. The polymer structure, electrochemical and thermal properties were characterized by various instrumental methods. The new polymer is expected to undergo electropolymerization leading to crosslinked polymers having conjugated segments in both main- and side-chain with enhanced conductivities and helical tunnels in the structure. Further studies in this line are now in progress.

## Experimental Section

### Materials

3-Thiophenecarboxylic acid % 99 (Acros), propargyl bromide solution in toluene ∼ %80 (Fluka), te-trabutylammonium bromide (+ %99) (Acros), (bicyclo[2,2,1]hepta-2,5-diene)chlororhodium(I) dimer ([(nbd)RhCl]^2^)≥ %98 (Fluka), diisopropylamine ≥ % 99 (Merck), triethylamine ≥ % 99.5 (Aldrich), were purchased and used as received. Solvents used for polymerization were purified before usage by the standard drying and distillation procedures.

### Characterization

The molecular weights of polymers were measured by GPC at 30 °C with an Agilent instrument (Model 1100) consisting of a pump, refractive index and UV detectors and four Waters Styragel columns (HR 5E, HR 4E, HR 3, and HR 2) eluent THF, flow rate of 0.3 mL/min and calibrated with polystyrene standards. Toluene was used as an internal standard. Data analyses were performed with PL caliber Software. ^1^H NMR spectra were recorded on a Bruker 250 Mhz spectrometer using CDCl_3_ as solvent and tetramethylsilane as the internal standard. FT-IR spectra were measured on Perkin-Elmer FT-IR Spectrum One spectrometer. Thermal gravimetric analysis (TGA) was performed on Perkin-Elmer Diamond TA/TGA with a heating rate of 10 °C min under nitrogen flow. Cylic voltammetry measurements were carried out using a Princeton Applied Research Model 2263. Cylic voltammetry in dichloromethane was performed using a 3-electrode cell (BASI model solid cell stand) with a polished 2mm sized Pt disc electrode as working electrode, a Pt wire counter electrode and an Ag/AgCl reference electrode, with a solution of polymer (6.6 g/l) and tetrabutylammonium perchlorate (TBAP,0.1, M) in CH_2_Cl_2_. All solutions were purged with nitrogen for at least 10 min before starting the measurements.UV-vis spectra were recorded on JASCO V-530 UV-vis spectro photometer.

### Monomer synthesis

In a 250 mL flask, of 3-thiophenecarboxylic acid (2.0 g, 15 mmol) was dissolved in 100 mL of 0.1 N NaOH. The mixture was heated at 50 °C until a clear solution was formed. To this solution, tetrabu-tylammonium bromide (0.50 g, 1.55 mmol) was added as a phase transfer catalyst. Then, a solution of propargylbromide (2.0 g, 17 mmol) in 20 mL of toluene was added portion wise. The mixture was kept stirring at 60 °C for 24 h. At the end of this period, it was cooled to afford solid. Additonally, the remaining toluene layer was separated and washed repeatedly with %2 NaOH (200 mL, 0.1 N) and with water. Evaporating toluene afforded extra solid.

### Polymerization

Polymerization was carried out under N_2_ atmosphere in a Schlenk tube equipped with a three-way stopcock. A typical polymerization procedure is as follows: A toluene solution (2.0 mL) of **1** (1 mmol) was added to a toluene solution (3.0 mL) of [(nbd)RhCl]_2_ (10^−3^ mmol) with co-catalyst diisopropylamine (10^−2^ mmol). Polymerization was carried out at 30 °C for 24 h.

## Figures and Tables

**Table 1.** Polymerization^a^ of acetylene-thiophene by Rh catalysts in conjunction^a^ with different co-catalysts.

Polymer	Co-catalyst	Yield (%)	*M*_n_^b^	*M*_w_/*M*_*n*_^b^
PAT-1	Triethylamine	12	2790	1.46
PAT-2	Diisopropylamine	20	4460	1.67
PAT-3	Butylamine	6	4690	1.33

a [M]_o_ = 0.2 M, [Rh]= 2 mM, [Co-catalyst] =20mM, 30 °C, 24 h;

b Determined by GPC according to polystyrene standards.

**Table 2.** Cylic voltammetry^a^ data and LUMO energy values of poly(acetylene thiophene) in dichloromethane. E_1/2_/V vs. Fc is the reduction potential versus ferrocene electrode (E_1/2_/V vs. Fc= (E_1/2_/V vs. Ag/AgCl)-(E_Fc_/V vs. Ag/AgCl)).

Electrode	E_pc_/V	E_pa_/V	E_1/2_/V vs. (Ag/AgCl)	E_Fc_/V vs.(Ag/AgCl)	E_1/2_/V vs. F_c_	LUMO (eV)
Pt disc	0.80	−0.33	0.24	0.47	0.71	4.09
	−0.61	−0.77	0.69	0.47	1.16	3.64

a Supporting electrolyte is 0.1 M tetrabutylammonium perchlorate (TBAP). [PAT-2] = 6.6 g/l.

**Table 3.** Thermal properties of polyacetylenes.

Polymer	T_5%_^a^ (°C)	T_10%_^b^ (°C)	T^c^_d max_ (°C)	Y_c_^d^ at 500°C (%)	Ref.
PAT-2	230	248	363	29	This work
Poly(phenylacetylene)	∼264	∼280	--	∼12	[35]

a T_5%_: The temperature for which the weight loss is 5%;

b T_10%_: The temperature for which the weight loss is 10%;

c T_d max:_ Maximum weight loss temperature;

d Y_c_: Char yields
